# Interactions of Borneol with DPPC Phospholipid Membranes: A Molecular Dynamics Simulation Study

**DOI:** 10.3390/ijms151120365

**Published:** 2014-11-06

**Authors:** Qianqian Yin, Xinyuan Shi, Haiou Ding, Xingxing Dai, Guang Wan, Yanjiang Qiao

**Affiliations:** 1 School of Traditional Chinese Medicine, Capital Medical University, No. 10 of Xitoutiao Outside Youanmen, Fengtai District, Beijing 100069, China; E-Mails: zmnh08830@126.com (Q.Y.); wungung91@163.com (G.W.); 2 Key Laboratory of TCM-Information Engineer, Beijing University of Chinese Medicine, No. 6 of Zhonghuan South Road, Wangjing, Chaoyang District, Beijing 100102, China; E-Mail: jolly_1987@163.com; 3 Civil Aviation General Hospital, No. 1 of Chaowai Takai A, Beijing 100123, China; E-Mail: dinghaiou890222@163.com

**Keywords:** borneol, traditional Chinese medicine (TCM), DPPC (1,2-dipalmitoylsn-glycero-3-phosphatidylcholine), molecular dynamics simulation, Martini

## Abstract

Borneol, known as a “guide” drug in traditional Chinese medicine, is widely used as a natural penetration enhancer in modern clinical applications. Despite a large number of experimental studies on borneol’s penetration enhancing effect, the molecular basis of its action on bio-membranes is still unclear. We carried out a series of coarse-grained molecular dynamics simulations with the borneol concentration ranging from 3.31% to 54.59% (*v*/*v*, lipid-free basis) to study the interactions of borneol with aDPPC(1,2-dipalmitoylsn-glycero-3-phosphatidylcholine) bilayer membrane, and the temperature effects were also considered. At concentrations below 21.89%, borneol’s presence only caused DPPC bilayer thinning and an increase in fluidity; A rise in temperature could promote the diffusing progress of borneol. When the concentration was 21.89% or above, inverted micelle-like structures were formed within the bilayer interior, which led to increased bilayer thickness, and an optimum temperature was found for the interaction of borneol with the DPPC bilayer membrane. These findings revealed that the choice of optimal concentration and temperature is critical for a given application in which borneol is used as a penetration enhancer. Our results not only clarify some molecular basis for borneol’s penetration enhancing effects, but also provide some guidance for the development and applications of new preparations containing borneol.

## 1. Introduction

Borneol is a monoterpenoid medicinal plant component that is widely used in clinical medicine. According to the theory of traditional Chinese medicine (TCM), borneol has pungent, bitter flavors and a cooling property, and it can act on the heart, spleen and lung channels. Due to its ability to clear pathogenic heat and clear the mind, borneol is widely used to treat febrile diseases, such as stroke, chest tightness, canker sores, eye disease, sore throats and some ear problems [1]. As a “guide” drug used in many traditional Chinese medicinal prescriptions, borneol not only acts as a drug, but it can also deliver other drugs to the action site. With the development of the modernization of TCM, numerous studies have been published on borneol in recent years covering many aspects, from manufacturing to application. Many researches have reported that borneol can be used as a natural penetration enhancer [2,3,4,5,6], due to its simple composition, reasonable price and the fact that it is less irritating than other chemical penetration enhancers. As previously reported, borneol can promote the penetration of both hydrophilic and lipophilic drugs, such as asiaticoside, ligustrazine, colchicine, cinnamic acid, Danshensu and tanshinone IIA. However, most of them have only focused on the penetration-enhancing behavior of borneol in *in vitro* tests, and the molecular mechanisms of borneol were rarely involved.

As an essential component of the body, the bio-membrane plays an important role in life activities, such as the transmembrane transport of certain substances. Most of borneol’s functions, especially the penetration enhancing effect, are based on bio-membranes. Therefore, carrying out systematic studies on the interactions between borneol and bio-membranes becomes very important, not only for understanding the mechanisms of borneol used as a penetration enhancer, but also for the development and clinical application of new preparations containing borneol. In recent decades, many experimental methods have been developed to investigate the effect of agents on various phospholipid bilayers, such as the electromagnetic spectrum [7], the microscopical technique [8], differential scanning calorimetry (DSC) [9], electron spin resonance (ESR) [10] and the electrochemical method [11].Extensive experimental efforts have elucidated the mechanisms of penetration enhancers to a certain degree, but the molecular details regarding borneol’s interaction with bio-membranes are still unclear. To clarify this issue, new techniques and methods need to be adopted urgently.

Molecular dynamics (MD) simulation is an effective and intuitional technique based on Newton’s equation of motion and is increasingly used in studying the interactions of agents with lipid bilayer systems. One can obtain larger temporal and spatial scales during a simulation by changing molecular resolution, which is generally known as mesoscopic or coarse-grain (CG) [12]. Similar to all-atom simulations, there are interactions between coarse-grained particles, and these interactions were often called coarse-grained force fields. The Martini model, developed by Marrink and his coworkers in 2007, is a special coarse-grained force field [13]. Martini was derived from atomistic models, especially for bonded interactions. The parameterizing process, based on experimental or atomistic data, in particular thermodynamic data, such as oil/water partitioning coefficients, provides a guarantee for its accuracy. Due to its portability and expansibility, Martini was widely used in many studies of biomolecules, such as lipids, polymers, proteins, carbohydrates, and so on [14,15,16,17,18]. DPPC, an amphoteric ionic compound, is the main component of the bio-membranes of mammals. Because its phase transition temperature (315 K) is close to physiological temperature, the DPPC bilayer system has been widely used as a model of bio-membranes in various studies [19]. Current results have shown that DPPC membranes can provide a reasonable explanation of the properties and functions of bio-membranes, such as elasticity, stability, permeability and the ability to respond to changes in environmental conditions [20]. Therefore, it can be used to investigate the interactions of agents with bio-membranes and to clarify their molecular mechanisms of adsorption and penetration.

Thus far, a number of MD simulation studies have been carried out to investigate the effects of some agents on membranes, involving DMSO, ethanol, oleic acid and dopamine [21,22]. The results yielded not only the key qualitative agreement with experiments, but also provided some supplementary information that can not be obtained by laboratory experiments. However, no MD simulation studies about the actions of borneol on bio-membranes have been reported yet. In this study, a series of coarse-grained MD simulations were carried out, aiming to provide some molecular insights into the interactions of borneol with bio-membranes to explain the mechanisms of borneol’s penetration enhancing effect, and then to provide some guidance for the clinical application of borneol as a penetration enhancer.

## 2. Results and Discussion

### 2.1. Concentration Effect

#### 2.1.1. Validity of DPPC (1,2-Dipalmitoylsn-glycero-3-phosphatidylcholine) Model Membrane

**Figure 1:**
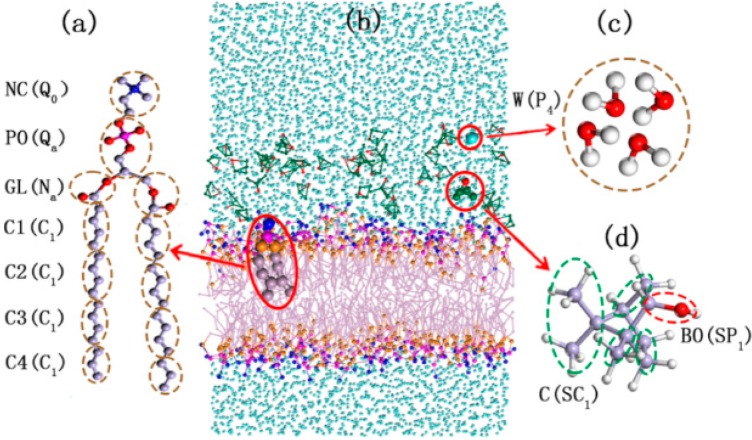
The structure and coarse-grained mapping for DPPC (1,2-dipalmitoylsn-glycero-3-phosphatidylcholine) (**a**); the DPPC membrane system with borneol in water (**b**); Water (**c**); and Borneol (**d**). Letters inside and outside of the parentheses are the name and Martini force field parameters of each coarse-grained beads. The green dots represent the carbon beads in CG borneol, the purple dots represent the PO (namely the CG site represents the phosphate groups in DPPC molecule), the pink dots represent the carbon beads in DPPC, the blue dots represent CG waters, the orange dots represent the GL beads (namely the glyceryl groups) of DPPC and red dots represent BO (the hydroxyl groups) beads of borneol.

Before discussing the effect of borneol, we validate the lipid bilayer model (Figure 1) employed in this study. The average area per lipid (APL) and bilayer thickness are two fundamental characteristics of the DPPC membrane. They define the lateral diffusion of lipids, the order of lipid alkyl chains to a large extent and can be measured accurately through experiments [20,23,24]. For the pure DPPC bilayer system, the APL from the simulations was found to be 68.17 Å^2^ (*T* = 345 K), which closely matches the experimental value of 67.10 Å^2^ (*T* = 338 K) [24] and the value of an earlier atomistic simulation study, 69.00 Å^2^ (*T* = 350 K) [25]. The bilayer thickness is defined as the peak-peak distance (D_H–H_) of NC (the CG site represents the choline groups in DPPC molecule) beads in opposite leaflets of the DPPC membrane according to the relative concentration profiles (Figure S1). The bilayer thickness of pure DPPC bilayers obtained from our study was 37.39 Å, which was consistent with the experimental value of 38.30 Å (*T* = 323 K) [20] and an earlier simulation study, 36.20 Å(*T* = 350 K) [25]. Clearly, the model employed in our work provides a reasonable description of the DPPC lipid bilayer.

#### 2.1.2. Morphology Perturbation of Bilayer Structure

**Figure 2:**
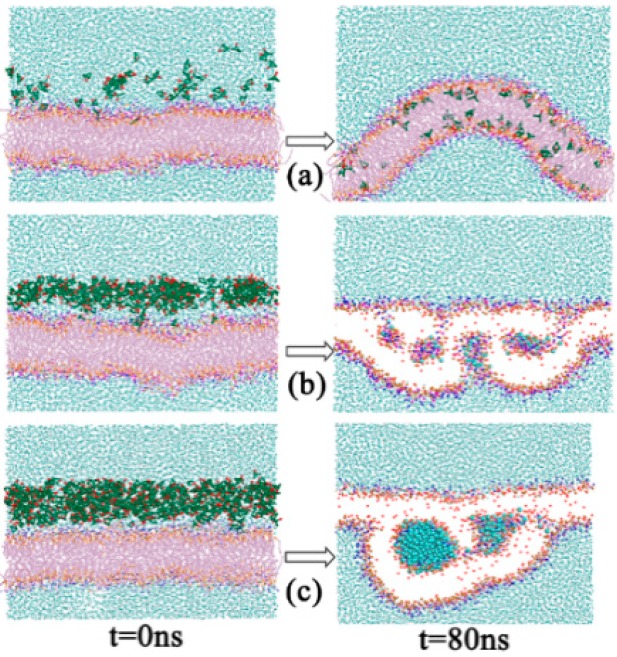
Snapshots of initial (*t* = 0 ns) and final (*t* = 80 ns) configurations of systems with different borneol concentration: 7.64% (**a**); 32.83% (**b**); and 54.59% (**c**). The green dots represent the carbon beads in CG borneol, the purple dots represent the PO (namely the CG site represents the phosphate groups in DPPC molecule), the pink dots represent the carbon beads in DPPC, the blue dots represent CG waters, the orange dots represent the GL beads (namely the glyceryl groups) of DPPC and red dots represent BO (the hydroxyl groups) beads of borneol. The DPPC lipid tails and beads in borneol, except BO are not shown, and water beads in thenon-bilayer structures within the bilayer interior are highlighted in 32.83% and 54.59% final configurations for the sake of brevity.

**Figure 3:**
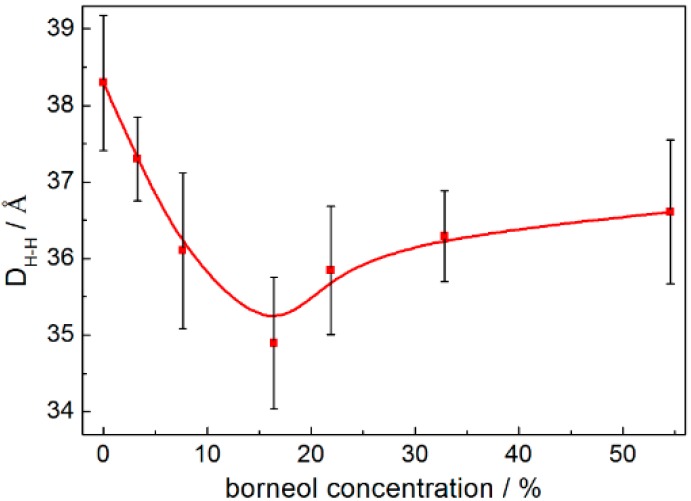
DPPC bilayer thickness of systems with different borneol concentrations: (**a**) 7.64%; (**b**) 32.83%; and (**c**) 54.59%. The error bars show the standard deviations that were estimated by block averaging.

The rotational time correlation function (RTCF) is not only a dynamic quantity depending on a time delay, t, but also an equilibrium property, obtained by the averaging of an equilibrium vector in the phase space. It is often used to evaluate if the systems have reached equilibrium within simulation times with APL. Here, we plotted the RTCF of the C3–C4 vector, which was the most fluid region in the DPPC bilayer membrane. The results are shown in Figure S2a, and it can be seen that the systems have reached equilibrium within 80 ns of simulation time, which was consistent with the APL profile (Figure S2b). Then, we studied the effects of borneol on DPPC bilayers. The results showed that adding a small amount of borneol molecules to a lipid bilayer had a dramatic effect on the membrane structure, and this action was significantly affected by the concentration of borneol (see Figure 2 and Figure S3). At concentrations of 16.42% and below, borneol caused different degrees of membrane fluctuation and lateral expansion, but the system remained in a whole bilayer structure. When the concentration was above 21.89%, borneol extracted some lipids from the upper layer to form inverted micelle-like structures, wrapping some water molecules into the bilayer interior (see Figure S4 for more information about the formation process of the non-bilayer structure), and the size of these inverted micelles enlarged with the concentration increasing. It is worth noting that these inverted micelle-like structures were long-lived, thus leading to irreversible changes in the membrane structure. Even after the removal of borneol molecules, the destroyed bilayer structure could not be recovered. Combined with the bilayer thickness results shown in Figure 3, at borneol concentrations below approximately 16.42%, the bilayer thickness decreased significantly with the borneol concentration increasing and reached the lowest value of 34.89 Å at 16.42%. This is consistent with some other interactions of small amphiphilic molecules with the lipid bilayer using both experiments [11,26,27] and simulation [25,28] methods from other teams’ study. This may be due to the fact that the membrane’s lateral expansion caused by borneol enlarged the space between lipids, and lipid tails have enough free volume to bend or fold on themselves, therefore resulting in a decreased DPPC bilayer thickness. When the borneol concentration was above 16.42%, the bilayer thickness increased with the borneol concentration further increasing. Because of the extraction effect of high concentration borneol on the upper layer DPPC lipids, the arrangement of DPPC molecules loosened and promoted the formation of non-bilayer structures, thereby leading to the increase of the bilayer thickness.

**Figure 4:**
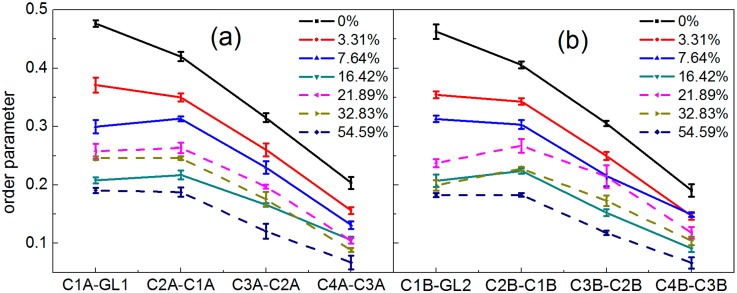
Lipid tail order parameters of systems with different borneol concentrations: (**a**) Chain A; and (**b**) Chain B. In all plots, solid and dashed lines represent a low and higher concentration range, respectively. The error bars show the standard deviations that were estimated by block averaging.

The order of parameters of the alkyl chains provides a measure of the alignment of DPPC in the bilayer. Here, the order of parameter *S*_z_ was defined as:

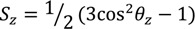
(1)
where *θ*_z_ is the angle between the *z*-axis (the bilayer normal) of the simulation box and the orientation vector of each bond. The results are shown in Figure 4. There appears to be more order near glyceride groups, and independent of a low concentration range (below 21.89%) or a higher concentration range (above 21.89%), the lipid tail order parameters decreased with the concentration increasing, suggesting that the penetration of borneol molecules caused the DPPC bilayer to be more disordered in the hydrophobic region.

#### 2.1.3. Configuration Changes of DPPC Molecules

**Figure 5:**
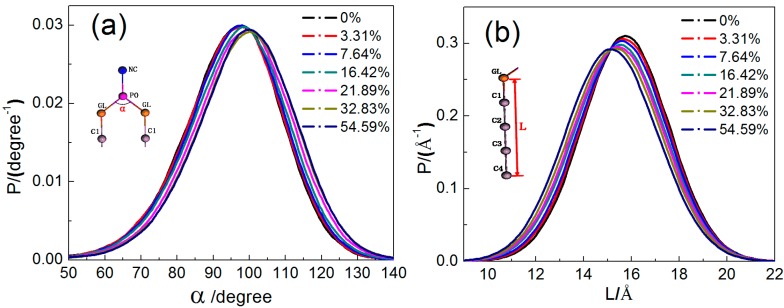
Distribution of the GL-PO-GL bond angle (**a**) and lipid alkyl chain length (**b**) in DPPC bilayer systems with different borneol concentration.

To study the effects of borneol on the structural and dynamic properties of the DPPC bilayer membrane, the configuration changes of DPPC molecules should be considered. Here, we analyzed the distribution of the angle α between two alkyl chains (see Figure 5a) and the length L of alkyl chains (see Figure 5b). It can be seen that all curves in both the angle and length distribution profiles are similar in shape with peak values located around 100° and 16 Å, respectively. With the increase in borneol concentration, the angle distribution curves shifted to the right, while the alkyl chain length distribution profiles shifted to the left. This indicates that the angle enlarged, while the lipid tail length decreased, which rationalizes the lateral expansion and thinning effect of borneol on the DPPC bilayer membrane.

#### 2.1.4. Distribution of Borneol within the DPPC Bilayer

From Figure 2, we can also see that the borneol molecules readily penetrate into the bilayer and occupy a position just beneath the lipid head groups with their hydroxyl groups (namely BO beads, represented as red dots in Figure 2) oriented towards the lipid head groups. This suggests that although borneol does not interact favorably with either of the DPPC hydrophilic head groups or the hydrophobic lipid tails, it has greater affinity with the head groups because of its amphiphilicity. This can also be seen through the relative concentration profiles (see Figure S1) of DPPC bilayer systems with different borneol concentrations. It can be seen that borneol molecules distribute mostly closest to the glyceride groups (namely GL beads), which is the most ordered region in the DPPC bilayer and which provides the main barrier to the permeation of small molecules [29]. In this region, borneol molecules act as spacers between lipid head groups and disrupt the intermolecular interactions, thus making the bilayer floppier and easier to fluctuate.

#### 2.1.5. Dynamical Properties

Mean square displacement (MSD) is the most common measure of the spatial extent of random motion. For a system at equilibrium, particles will tend to diffuse away from their original location because of inter particle collisions. The MSD of the particles with respect to their original position is obtained as the second moment of their distribution at *t* > 0 and is related to the diffusion coefficient (D) as follows:

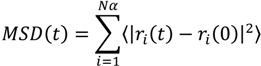
(2)

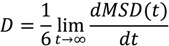
(3)
where *r*_i_ is the position vector of the *i*-th particle, and the angular brackets denote an ensemble average [30].

**Figure 6:**
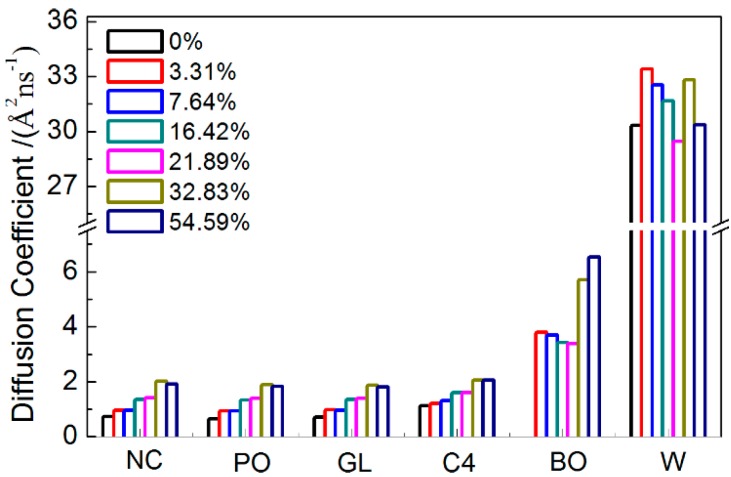
Diffusion coefficient of each type beads in systems with different borneol concentrations.

In lipid membrane systems, the mean square displacement and diffusion coefficient are often used to describe the fluidity of the membrane itself and the permeation of small agents. Here, we calculated the diffusion coefficient of each type of bead along a normal bilayer (see Figure 6). It can be seen that, for beads in the DPPC, the diffusion coefficient increased with increasing borneol concentration, thereby suggesting that borneol’s penetration and accumulation fluidized the DPPC bilayer membrane. While for borneol, the diffusion coefficient decreased first and then increased with increasing concentration. This may be due to the fact that borneol molecules tend to aggregate themselves in aqueous solution (see Figure S5), and concentration increases could reinforce this effect, therefore hindering the penetrating rate of borneol into the DPPC membrane. However, when borneol’s concentration was at 21.89% and above, the formation of non-bilayer structures caused a significant number of water molecules to spread into the bilayer interior, which decreased the ordering of the DPPC alkyl chains significantly, thus increasing the penetrating rate of borneol.

#### 2.1.6. A Temporary Water Pore

**Figure 7:**
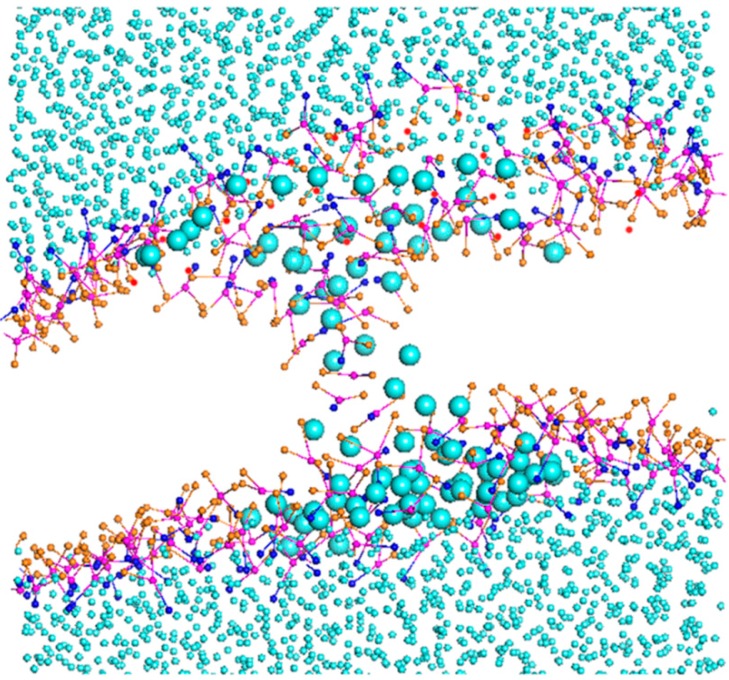
Temporary water pore formation in a tensionless DPPC bilayer with 7.64% borneol during 1360~6040 ps. The green dots represent thecarbon beads in CG borneol, the purple dots represent the PO (namely the CG site represents the phosphate groups in DPPC molecule), the pink dots represent the carbon beads in DPPC, the blue dots represent CG waters, the orange dots represent the GL beads (namely the glyceryl groups) of DPPC and red dots represent BO (the hydroxyl groups) beads of borneol. The DPPC lipid tails and beads in borneol except BO are not shown, and water beads in the pore are highlighted for the sake of clarity.

It is also worth noting that a temporary water pore was formed spontaneously in the DPPC bilayer with 7.64% borneol during 1360~6040 ps (see Figure 7), as was observed in Notman’s study of DMSO interacting with the DPPC bilayer [31]. This may be due to the lateral loosening of the DPPC bilayer, which was caused by borneol’s penetration into the bilayer interior, making it easier for water to permeate into the bilayer.

### 2.2. Temperature Effect

To investigate the effects of temperature on the interactions of borneol with the DPPC bilayer membrane, two systems with 7.64% and 32.83% borneol were selected to run dynamics at different simulation temperatures. Given that the phase transition temperature of DPPC is 315 K, we set it at two points higher and lower than that, namely at 285, 305, 325 and 345 K.

#### 2.2.1. Interactions of Borneol with the DPPC Bilayer

**Figure 8:**
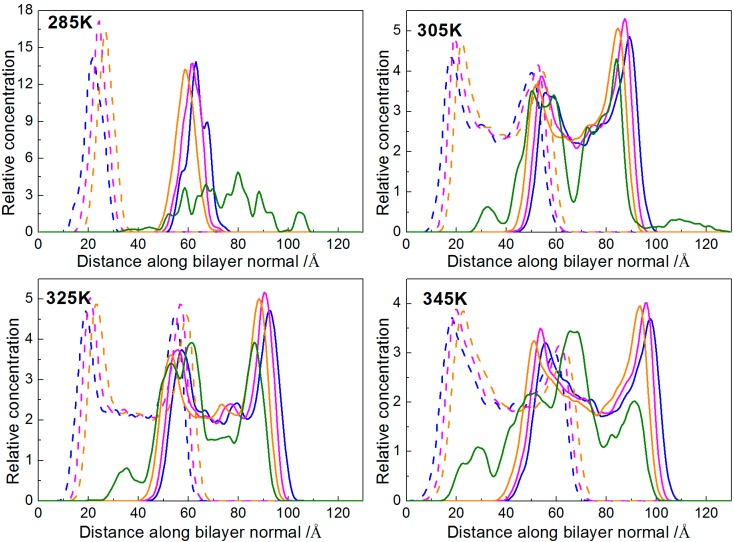
Relative concentration profiles of the DPPC bilayer system with 7.64% borneol at different temperatures. In all plots, blue, pink and orange lines represent the profiles of NC, PO and GL beads in DPPC bilayer, while green lines represent the BO beads of borneol. The solid and dash lines denote opposite sides of the bilayer.

The relative concentration profile is a mass density (or electron density) distribution along the bilayer normal. Here, we calculated the mass density distribution of beads NC, PO, and GL respectively, with the bilayer normal along the *z*-axis. In order to avoid accidental results, we analyzed the distribution of the last 200 frames to obtain the average distribution of them. The relative concentration profiles of each system at different temperatures are shown in Figure 8 and Figure S6. It can be seen that, for the system with 7.64% borneol, the DPPC membranes could keep complete bilayer structures with different degrees of thinning at four simulation temperatures. In addition, with rising temperatures, there were more borneol molecules that penetrated into the DPPC bilayer.

Radial distribution functions (RDFs) provide a measure of the probability that, given the reference particle α at the origin of an arbitrary reference frame, there will be a particle β with its center located in a spherical shell of infinitesimal thickness at a distance, *r*, from the reference particle α. Note that the reference particle α and the particle β can be of the same or of different types [30]. According to Hansen and McDonald [32], *g*_αβ_(*r*) is defined as:

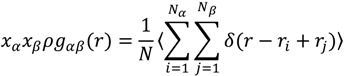
(4)
where *x_i_* is the mole fraction of particle type *i*, *N_i_* is the number of particles of type *i*, *N* is the total number of particles, *ρ* is the overall number density and angular brackets represent the ensemble average.

**Figure 9:**
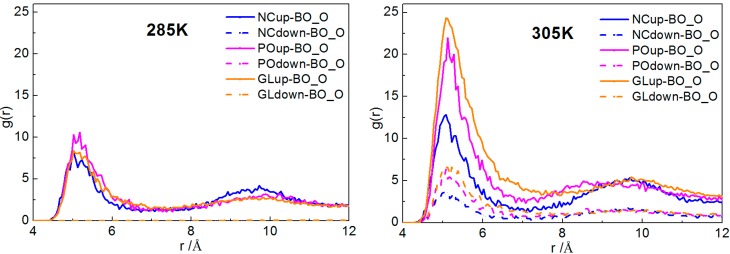

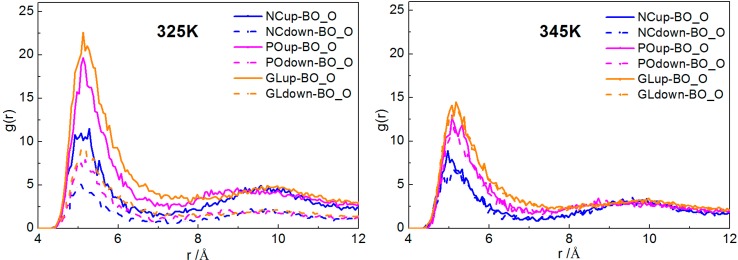
Radial distribution functions (RDFs) of the system with 7.64% borneol at different temperatures. In all plots, blue, pink and orange lines represent the profiles of NC-BO, PO-BO and GL-BO beads. The solid and dashed lines denote the opposite sides of the bilayer, and the data of *r* > 12 Å are not shown, for the sake of brevity.

**Figure 10:**
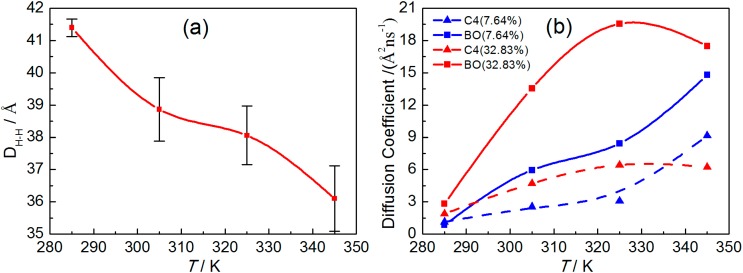
The DPPC bilayer thickness of the systems with 7.64% borneol at different temperatures (**a**) and the diffusion coefficient of C4 (triangle) and BO (square) beads in systems with 7.64% (blue lines) and 32.83% (red lines) borneol at different temperatures (**b**). The error bars show the standard deviations that were estimated by block averaging.

Here, we plotted the RDFs of NC-BO, PO-BO and GL-BO beads at different temperatures. At each temperature, in order to avoid accidental results, we analyzed the RDFs of the last 200 frames to obtain the average distribution of them, shown in Figure 9. It can be seen that, at 285 K, all the RDFs of NC-BO, PO-BO and GL-BO beads in the lower layers were zero, and all of the peak values in the upper layers were lower than the other three temperatures. This suggested that fewer borneol molecules penetrated into the bilayer and just distributed into the upper layer. Even more remarkable, the peak value of the PO-BO curve was higher than both the GL-BO and NC-BO curves in the upper layer, indicating that the borneol molecules that had penetrated into the bilayer accumulated at the lipid/water interface region. This was due to the fact that the motion of borneol molecules was restricted by the freezing effect of water to a great extent. When the temperature was raised to 305 K, almost all of borneol molecules penetrated into the bilayer interior and accumulated close to glyceride groups (namely GL beads). With the temperature further raised, all borneol molecules penetrated into the bilayer interior and tended to diffuse the equilibrium in opposite sides of the DPPC bilayer. The bilayer thickness of the system with 7.64% borneol was also analyzed at different temperatures, as shown in Figure 10a. With the temperature raised, the bilayer thickness decreased dramatically. This was due to the fact that high temperatures disrupted the interaction between head groups and loosened the lateral arrangements of DPPC molecules, which ensured that the DPPC lipid tails had more free volume for bending and folding.

For a system with 32.83% borneol (Figure S6 and S7), the DPPC bilayer structure had significant changes at different temperatures. At 285 K, most borneol molecules could penetrate into the bilayer, and there were also borneol molecules that existed in the lower layer. With raised temperatures, some borneol molecules distributed close to glyceride groups, while other borneol molecules extracted lipids from the upper layer and formed non-bilayer structures. With time, these non-bilayer structures were wrapped into the bilayer interior with some water molecules and, finally, formed invert micelle structures.

#### 2.2.2. Dynamical Properties

Finally, we paid attention to the fluidity of the DPPC bilayer and the diffusion property of borneol at different temperatures. The diffusion coefficient of C4 bead in DPPC and the BO bead in borneol were calculated according to MSD profiles (Figure S8), shown in Figure 10b. We found that the diffusion coefficients of both EC and BO beads increased with increasing temperatures in systems with 7.64% borneol. However, for systems with 32.83% borneol, when the temperature was below 325 K, both diffusion coefficients increased with rising temperatures, while a further increase in temperature resulted in the decrease of the diffusion coefficient. This suggested that at a low borneol concentration, a rise in temperature could fluidize the DPPC bilayer and promote the penetrating progress of borneol. However, at high borneol concentrations, there was an optimum temperature for the interactions of borneol with the DPPC bilayer, which may be due to the fact that the formation of inverted micelle-like structures within the bilayer interior affected the temperature effects.

## 3. Simulation Details

All of the MD simulations in this work were carried out in a coarse-grained method, which enables longer time and length scales to be accessed within the simulations, because of its high computation efficiency. The DPPC and borneol molecular topologies used in this paper were obtained from the PubMed database and preprocessed using the Discover Module in the Materials Studio 5.5 (Accelrys Inc., Cambridge, UK) software package [30], the same software package on whichall of the simulations in this study were performed. After the energy minimization preprocess, the molecules were coarse-grained and parameterized in the mesocite module, which is a mesoscopic module integrated in Materials Studio, mainly based on the coarse-grained force field. According to the principle of the Martini force field, for aliphatic compounds, *i.e.*, DPPC, on average, four heavy atoms and the hydrogen atoms attached to them are represented by a single interaction site, namely one bead, while 2 or 3 to 1 mapping was used for ring structures, *i.e.*, borneol, and four atomistic water molecules are mapped to one Martini site. It is worth noting that because of the larger CG particle sizes, the underlying energy landscape in CG is much smoother than that in all atomistic models; therefore, the dynamics observed with CG models is faster. According to Marrink’s study, the effective time sampled using the Martini model was found to be two-to-tenfold larger compared with the atomistic model, such as the water permeation rates across the DPPC bilayer and lipid lateral diffusion rates, and the aggregation of lipids into vesicles are found to be consistent with experimental results after scaling the time by a factor of four [33,34,35]. Thus, the simulation times reported in this paper are effective times, unless explicitly stated. To improve the computational efficiency further, a standard bead mass *m* = 72 amu (corresponding to four atomistic water molecules) is typically used for aliphatic compounds and 45 amu for ring structures. The details of coarse-grained mapping and force field parameterizing for each molecule are given in Figure 1. The Martini force field parameter for DPPC was obtained from Marrink’s study. Because no Martini force field parameter for borneol had been reported before, we parameterized it using a method provided on the Martini website according to the general Martini force field [13]. The results showed that the coarse-grained Martini borneol could mimic both the structural and thermodynamic properties compared with all atomistic simulation and experimental results (See the Supplementary Materials for more details). Then, bilayer systems with different borneol concentrations in water were built in the Mesocite Module: A pure DPPC (362 lipids) bilayer in water (A), System A containing 26 (B), 60 (C), 129 (D), 172 (E), 258 (F) and 429 (G) borneol molecules added, which correspond to a volume percent (*v*%, top water basis) of borneol in the bilayer of 3.31%, 7.64%, 16.42%, 21.89%, 32.83% and 54.59%, respectively. Considering the transport through the membrane of agents from the outside of the cell to the inside, the borneol molecules were just randomly placed in the top water layer (3929 water molecules) with some water molecules being replaced by borneol molecules, and another water layer (1100 water molecules) was added on top to prevent borneol from diffusing through the opposite water layer (3929 water molecules), because of the three-dimensional periodic boundaries. Table 1 summarizes the composition of systems used in this work.

**Table 1:** Composition of the systems studied in the present work.

Systems	DPPC	Bottom Water	Top Water		Extra Water	*v*%	mol %
			Water	Borneol			
A	362	3929	3929	0	1100	0	0
B	362	3929	3799	26	1100	3.31	2.09
C	362	3929	3629	60	1100	7.64	4.91
D	362	3929	3284	129	1100	16.42	10.93
E	362	3929	3069	172	1100	21.89	14.90
F	362	3929	2639	258	1100	32.83	23.40
G	362	3929	1784	429	1100	54.59	42.91

Before the production dynamic simulations, four smart (the smart algorithm is a cascade method accessed in Materials Studio; it starts with thesteepest descent method [36], followed by the conjugate gradient method [37], and ends with a Newton method [38]) energy minimization calculations and one 1600-ps equilibrium dynamics under the NPT (constant particle number, constant system pressure and constant temperature) ensemble were performed on each system. Temperature was controlled at 273 K by the Berendsen thermostat with a time constant of 1.0 ps [39]. Pressure was coupled isotropically by the Berendsen barostat with a time constant of 1.0 ps and a reference pressure of 1 bar [39]. Three-dimensional periodic boundary conditions were applied. The coulomb interactions were controlled with the Ewald method [40,41], while the van der Waals interactions were controlled with group-based cutoff method [42], both of the cutoffs were set to 15.5 Å. Additionally, the time step was set to 10 fs to prevent the dynamics from crashing. After equilibrium, 80 ns dynamics was performed on each system. Considering the cost of computation, it is worth noting that before performing the systematic studies, we have taken the DPPC bilayer system with 21.89% borneol molecules as an example to select a reasonable simulation time, which could reduce the cost as much as possible while maintaining the simulation quality (see Figures S11 and S12 for more information). The temperature was controlled by a nose-hoover thermostat with a time constant of 1.0 ps [43,44,45]. When studying the effects of borneol’s concentration on the bilayer, the temperature was set to 345 K, which was higher than the phase transition temperature (315 K) of DPPC, to ensure that the bilayer was staying in the liquid crystalline phase, which agreed with the physiological state. According to our pre-experiment results (See Figure S13 for more information), this temperature was proven to be still higher than the phase transition temperature of the Martini model. The time step was set to 30 fs; other parameters were set the same as used in the equilibrium dynamics, and the last 24 ns were used for analysis. Repeated simulations with different random starting conditions show the same behavior. To explore whether the destroyed bilayer structure in systems with borneol concentration of 32.83% and 54.59% could be recovered, we performed an extraneous 36 ns simulation on the two systems with all borneol molecules removed.

## 4. Conclusions

Here, we presented a series of coarse-grained molecular dynamics simulations to investigate the interactions of borneol with the DPPC bilayer membrane. We systematically varied the borneol concentrations from 3% to 54.59% (*v*/*v*, lipid-free basis). Then, we took systems with 7.64% and 32.83% borneol as examples to investigate the effects of temperature.

The results demonstrate that the presence of borneol molecules at any concentration within the bilayer interior can fluidize the bilayer membrane. At low concentrations, borneol caused different degrees of membrane fluctuation and lateral expansion with decreases in bilayer thickness. While at high concentrations, the formation of long-lived inverted micelle-like structures within the bilayer interior led to irreversible changes in the membrane structure. The effects of temperature are also influenced by borneol’s concentration. At low concentrations, a rise in the temperature always promoted the penetrating progress of borneol, while at high concentrations, there was an optimum temperature for the interactions of borneol with the DPPC bilayer membrane.

These findings are in agreement with the interactions between ethanol and the POPC bilayer membrane studied by experiment [46] and simulation [28] methods. Thus far, studies have found that borneol can be used as a natural penetration enhancer for both hydrophilic and lipophilic drugs, especially the latter, whose mechanisms are more easily understood. However, the mechanisms of borneol’s penetration enhancing effect on hydrophilic drugs have not yet been devised. The thinning and expansion effects of borneol on the DPPC membrane may be beneficial for the penetration progress of lipophilic drugs. In addition, the observation of a temporary water pore at 7.64% and inverted micelle-like structures at high concentrations in our simulations may provide some guidance to clarify this puzzle. These non-bilayer structures may provide a delivery system for hydrophilic drugs and ions. However, we are not ruling out that the non-bilayer structures are linked to borneol’s toxicity at high concentrations, which needs further study.

In conclusion, a rise in the concentration and temperature within a restricted range could improve the penetration enhancing property of borneol, which emphasizes the importance of identifying an optimal concentration and temperature in practical applications. At this point, we have not only provided some molecular basis for borneol’s penetration enhancing effects, but also some guidance for development and applications of new preparations with borneol. In addition, we adopted a new technology based on computer simulation to investigate the mechanisms of “yaofuheyi” (yaofuheyi means that an agent in a preparation not only has the effect of the drug, but also can be used as an excipient) in Traditional Chinese Medicine, which can promote the modernization progress of TCM.

## Supplementary Materials

Supplementary materials can be found at http://www.mdpi.com/1422-0067/15/11/20365/s1.
